# A case report of constrictive pericarditis following COVID-19 vaccination

**DOI:** 10.1093/ehjcr/ytad540

**Published:** 2023-11-06

**Authors:** Eric Bain, Maya Guglin

**Affiliations:** Department of Internal Medicine, Indiana University School of Medicine, 635 Barnhill Drive, Van Nuys Medical Science Building 116, Indianapolis, IN 46202, USA; Krannert Institute of Cardiology, Indiana University School of Medicine, Indianapolis, IN, USA

**Keywords:** COVID-19, Vaccine, Constrictive, Pericarditis, Pericardiectomy, Diastolic heart failure, Case report

## Abstract

**Background:**

COVID-19 infection and the COVID-19 vaccines have been associated with rare cases of pericarditis. We present a case of constrictive pericarditis (CP) following the vaccine.

**Case summary:**

A 19-year-old healthy male started having progressive abdominal pain, emesis, dyspnoea, and pleuritic chest pain 2 weeks after the second dose of Pfizer vaccine. Computed tomography angiography chest revealed bilateral pleural effusions and pericardial thickening with effusion. Cardiac catheterization showed ventricular interdependence. Cardiac magnetic resonance (CMR) showed septal bounce and left ventricular tethering suggestive of CP. A total pericardiectomy was performed with significant symptom improvement. Pathology showed chronic fibrosis without amyloid, iron deposits, or opportunistic infections. Patient had Epstein–Barr Virus (EBV) viraemia 825 IU/mL and histoplasmosis complement-fixation positive with negative serum and urine antigen. Hypercoagulable panel and infectious workup were otherwise negative. The patient had resolution of cardiac symptoms at 3 months of follow-up.

**Discussion:**

The patient developed progressive symptoms within 2 weeks of his second Pfizer vaccine. Echocardiogram and CMR had classic signs of CP, and pericardial pathology confirmed fibrotic pericardium. The patient had no prior surgery, thoracic radiation, or bacterial infection. Epstein–Barr Virus viraemia was thought to be reactionary, and histoplasmosis complement likely represented chronic exposure. The timing of symptoms and negative multidisciplinary workup raises the suspicion for COVID vaccine–induced CP. The COVID vaccines benefits far exceed the risks, but complications still can occur. Practitioners should have a high index of suspicion to allow prompt diagnosis of CP.

**Learning points**

- To recognize constrictive pericarditis as a rare side effect of COVID-19 vaccination.
- To identify presenting symptoms of patients with constrictive pericarditis.

## Introduction

COVID-19 vaccination has been associated with rare cases of pericarditis. There are only a few case reports describing constrictive pericarditis (CP) following pericarditis from COVID-19 vaccination.^6–10^ We report a case of a 19-year-old male found to have CP requiring pericardiectomy following the second dose of the COVID-19 mRNA vaccine.

## Summary figure

**Table:** 

Timeline	Events
Day 0	Initial COVID vaccination administered
Week 3	Booster COVID vaccination administered
Week 5	Onset of nausea, vomiting, and epigastric pain
Months 1–4	Multiple emergency room visits with eventual referral to gastroenterology with new symptoms of diarrhoea
Month 5 (hospital Day 0)	Symptoms progressed with pleuritic chest pain and dry cough. Right upper quadrant (RUQ) ultrasound (US) showing right pleural effusion and ascites prompting computed tomography angiography chest with moderate to large pericardial effusion. Patient admitted to the hospital
Hospital Day 1	Patient started on non-steroidal anti-inflammatory drugs (NSAIDs)
Hospital Day 2	Right heart catheterization (RHC) consistent with constrictive pericarditis (CP) with ventricular interdependence
Hospital Day 8	Cardiac magnetic resonance imaging (MRI) with prominent septal bounce and apical tethering
Hospital Day 15	Pericardial biopsy showing only fibrous tissue and chronic inflammation. Infectious workup to date without clear cause of CP
Hospital Day 26	Patient remained symptomatic despite treatment. Decision made to proceed with total pericardiectomy
Hospital Day 31	Patient discharged without post-surgical complications
Month 9	Follow-up transthoracic echocardiogram (TTE) with normal diastolic and systolic function. Patient reportedly doing well without new symptoms

## Case presentation

**Figure 1:**
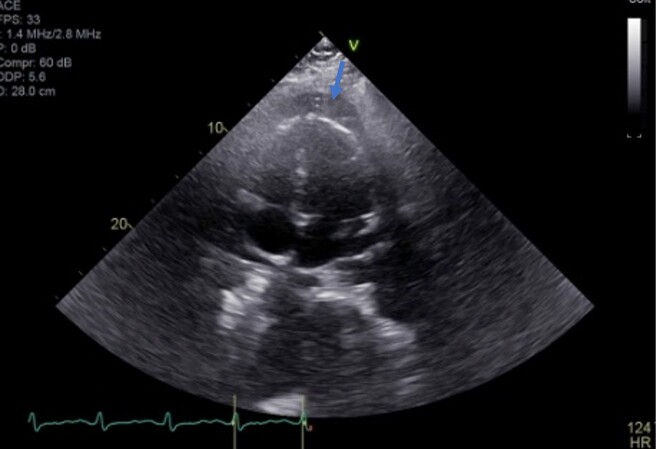
Transthoracic echocardiogram four-chamber view demonstrating pericardial effusion (arrow).

A 19-year-old previously healthy male reported a 4-month history of abdominal pain, nausea, and vomiting beginning 2 weeks after his second dose of the mRNA COVID-19 vaccine (BNT162b2). His booster COVID vaccine was administered 3 weeks after initial vaccine. He had no past medical history. He presented to his primary care physician and the emergency department (ED) on three separate occasions for these symptoms and was treated with antiacids and antiemetics. He was referred to gastroenterology and underwent an outpatient right upper quadrant (RUQ) ultrasound (US) which demonstrated cholelithiasis, ascites, and a right pleural effusion. Laboratory tests were obtained at that time and were significant for a mildly elevated total bilirubin (3.9 mg/dL), anaemia (Hgb 12.4 g/dL), elevated erythrocyte sedimentation rate (22 mm/h), and elevated C-reactive protein (7.8 mg/dL). Chest radiograph was obtained demonstrating borderline enlarged cardiac silhouette. His symptoms progressed to include diarrhoea, shortness of breath, and pleuritic chest pain prompting him to return to the ER. Vitals were significant for tachypnoea (26 breaths/min) and tachycardia (123 beats/min). The physical exam was negative for jugular vein distension, pericardial rub, and extremity oedema. A 12-lead electrocardiogram showed sinus tachycardia with low-voltage QRS complexes. Computer tomography angiography of the chest demonstrated pericardial thickening and effusion with moderate bilateral pleural effusions. Echocardiogram was technically limited, but demonstrated left ventricular ejection fraction (LVEF) of 55%, mild right ventricular dilatation, and a moderate sized ‘jelly-like’ pericardial effusion and E/A ratio of 2.33 (*Figure 1*). A mild septal bounce was appreciated. Infectious workup was notable for histoplasmosis complement fixation positivity with negative serum and urine antigen and positive IgM mycoplasma with negative polymerase chain reaction (PCR) in addition to low-grade Epstein–Barr viraemia (EBV) (827 IU/mL). Infectious disease suspected EBV viraemia to be reactivation with repeat titre resulting negative. Additional workup including inflammatory panel, hypercoagulable panel, and gastrointestinal pathogen panel was negative. He underwent a left thoracentesis which demonstrated transudative fluid negative for malignancy and acid-fast bacteria.

Clinical and imaging findings were suggestive of elevated right heart pressures and diastolic heart failure (HF). Differential considerations initially included cardiac tamponade, effusive–constrictive pericarditis, severe tricuspid regurgitation, pulmonary hypertension, and restrictive cardiomyopathy.

**Figure 2:**
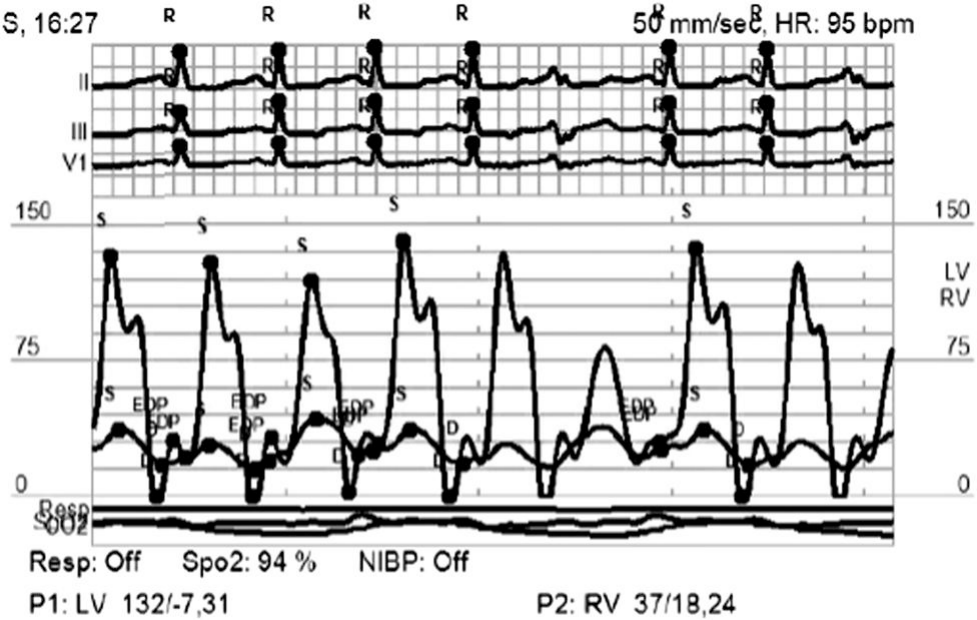
Simultaneous left ventricular (LV)–right ventricular (RV) pressure tracings demonstrating increased filling of the right ventricle and decreased filling of the left ventricle during inspiration compatible with ventricular interdependence.

**Figure 3:**
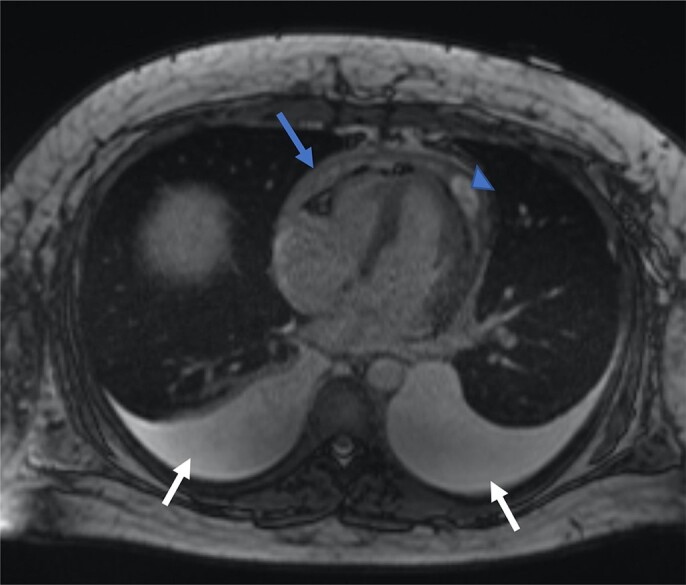
Cardiac magnetic resonance true fast imaging with steady-state free precession (TRUFI) with pericardial thickening (dark arrow) and small loculated pericardial effusion (dark arrowhead). Moderate bilateral pleural effusions (white arrows).

**Figure 4:**
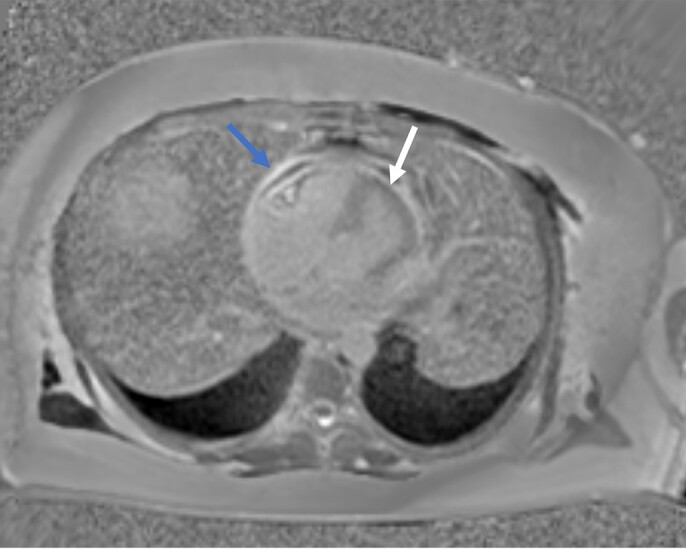
Delayed post-contrast cardiac magnetic resonance four-chamber phase-sensitive inversion recovery (PSIR) demonstrating pericardial thickening with delayed pericardial enhancement of the visceral (white arrow) and parietal layers (dark arrow).

Further cardiac and gastrointestinal workup was performed. Colonoscopy and endoscopy with duodenal biopsy were unremarkable. Cardiac catheterization was suggestive of constrictive physiology with near equalization of the left ventricular end-diastolic pressure (LVEDP 26 mmHg) and pulmonary capillary wedge pressure (PCWP 28 mmHg) during inspiration, mild pulmonary hypertension (pulmonary artery 32 mmHg), and ventricular interdependence. (*Figure 2*). Cardiac magnetic resonance demonstrated diffuse pericardial thickening and delayed gadolinium enhancement of the visceral and parietal surfaces. The parietal pericardium measured up to 5.8 mm. There was a small anterior loculated pericardial effusion. A focal region of pericardial tethering was seen along the inferior wall of the left ventricle. Cine imaging demonstrated ventricular interdependence. (*Figures 3* and *4*). There was no delayed enhancement of the myocardium or myocardial oedema to suggest acute myocarditis.

**Figure 5:**
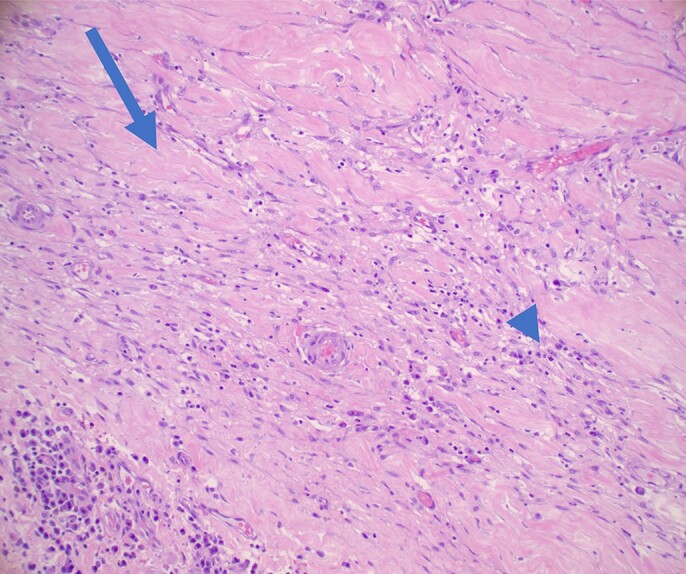
Haematoxylin and eosin 40× stain of pericardial tissue–fibrous tissue (arrow) with patchy mild chronic inflammation (arrowhead).

He was treated with non-steroidal anti-inflammatory drugs (NSAIDS) and colchicine for pericarditis. Diuretics were used to reduce oedema and elevated venous pressures. Pericardial biopsy was performed with non-specific chronic inflammatory changes (*Figure 5*). He remained hospitalized with continued chest pain, peripheral oedema, and tachycardia despite 20 days of ibuprofen and 18 days of colchicine. The patient had been symptomatic for nearly 6 months to this point. Cardiothoracic surgery was consulted, and the decision was made to pursue total pericardiectomy. Pericardial pathology showed fibrotic tissue with mild to moderate patchy chronic inflammation and haemorrhage. Haematoxylin and eosin slides of the endocardium revealed no evidence of amyloid, iron deposition, or opportunistic infection. The post-surgical course was unremarkable, and he was discharged 5 days post-operatively.

Follow-up included repeat echocardiogram at 2 months post-operative which demonstrated a normal LVEF (55%) and normal diastolic filling pressures. At recent 3-month follow-up, the patient denied new cardiac symptoms. Repeat infectious labs are without evidence of active histoplasmosis or EBV.

## Discussion

Constrictive pericarditis is an inflammatory condition which results in pericardial fibrosis and loss of pericardial compliance eventually causing diastolic HF. It is estimated 9% of acute pericarditis cases develop into CP with a mean time between symptom onset and diagnosis of 17 months.^1,2^ The wide time interval and insidious onset of symptoms makes diagnosis challenging. The most common cause of CP is idiopathic/viral and often a clear source cannot be identified.^2^

Rare cases of myocarditis and pericarditis have been reported following mRNA COVID-19 vaccination. This most frequently has been seen in a younger male demographic (18–25 years old) following the second vaccine dose.^3–5^ An increased risk has also been seen with short interval (<30 days) between the first and second vaccine administration and with mRNA-1273 (Moderna) compared with BNT162b2 (Pfizer-BioNTech Comirnaty).^4,5^ Very few cases have been documented in which a patient developed acute pericarditis and subsequent CP related to COVID-19 vaccine,^6–10^ but the reported cases were either milder^6,7^ or occurred in the setting of another systemic inflammatory disease which can cause CP by itself.^8^

Our patient fit the common demographics seen with COVID-19–related pericarditis; he is a young adult male and experienced symptoms following his second vaccine dose after a short (3 weeks) time interval between the two doses. An extensive workup was performed without evidence of infectious, inflammatory/autoimmune, or malignant sources. He had no prior history of thoracic surgery or radiation. Constrictive pericarditis secondary to mycoplasma or histoplasmosis was considered due to the initial positive IgM and complement factor respectively; however, the negative PCR and urine antigen suggested these were chronic exposures. Epstein–Barr Virus viraemia is a known entity in the setting of impaired cellular function, and in this case, the low level of IgM positivity was favoured to be reactivation. The timing of symptom onset and negative multidisciplinary workup raises the suspicion for COVID-19 vaccine–induced CP.

The long-term impact of vaccine-related CP and associated morbidity remain unknown. It is likely that more cases exist that have not been reported due to mild symptoms and underdiagnosis. Ongoing review of vaccination programmes should be performed to better assess the risk and natural history.

## Conclusion

The mRNA COVID vaccines have reduced morbidity and mortality associated with COVID-19 infection, but complications do occur. This case adds to existing reports suggesting that CP may be a rare side effect of vaccination. Continued analysis of ongoing vaccination programmes should be considered. At present, practitioners should be aware of this possible complication and have a high index of suspicion to allow prompt diagnosis.

## Supplementary Material

ytad540_Supplementary_Data

## Data Availability

All data are incorporated into the article and its online Supplementary material.

## Lead author biography

Dr Eric Bain is an internal medicine resident at Indiana University School of Medicine. He completed medical school at Indiana University. He’s currently pursuing a career in cardiology. His areas of interest include advanced HF and the integration of multimodality imaging.
