# Supplementary figures and images for: A case report of constrictive pericarditis following COVID-19 vaccination
Source: Eur Heart J Case Rep. 2023 Nov 6;7(11):ytad540. doi: 10.1093/ehjcr/ytad540 (PMC10656752; doi:10.1093/ehjcr/ytad540)

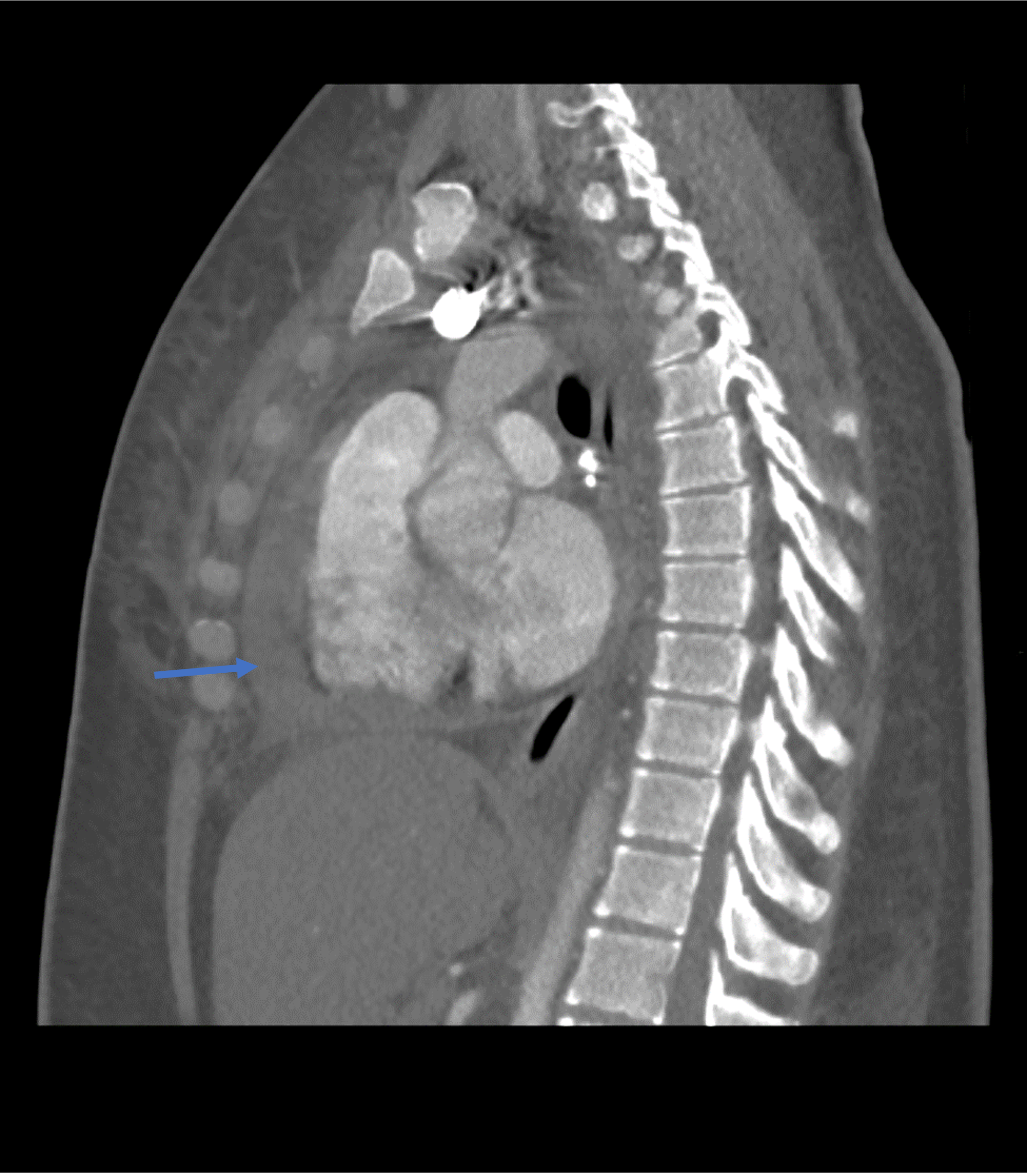

Supplement: ytad540_Supplementary_Data [file ytad540_supplementary_data.zip › Sup Fig 2.png]

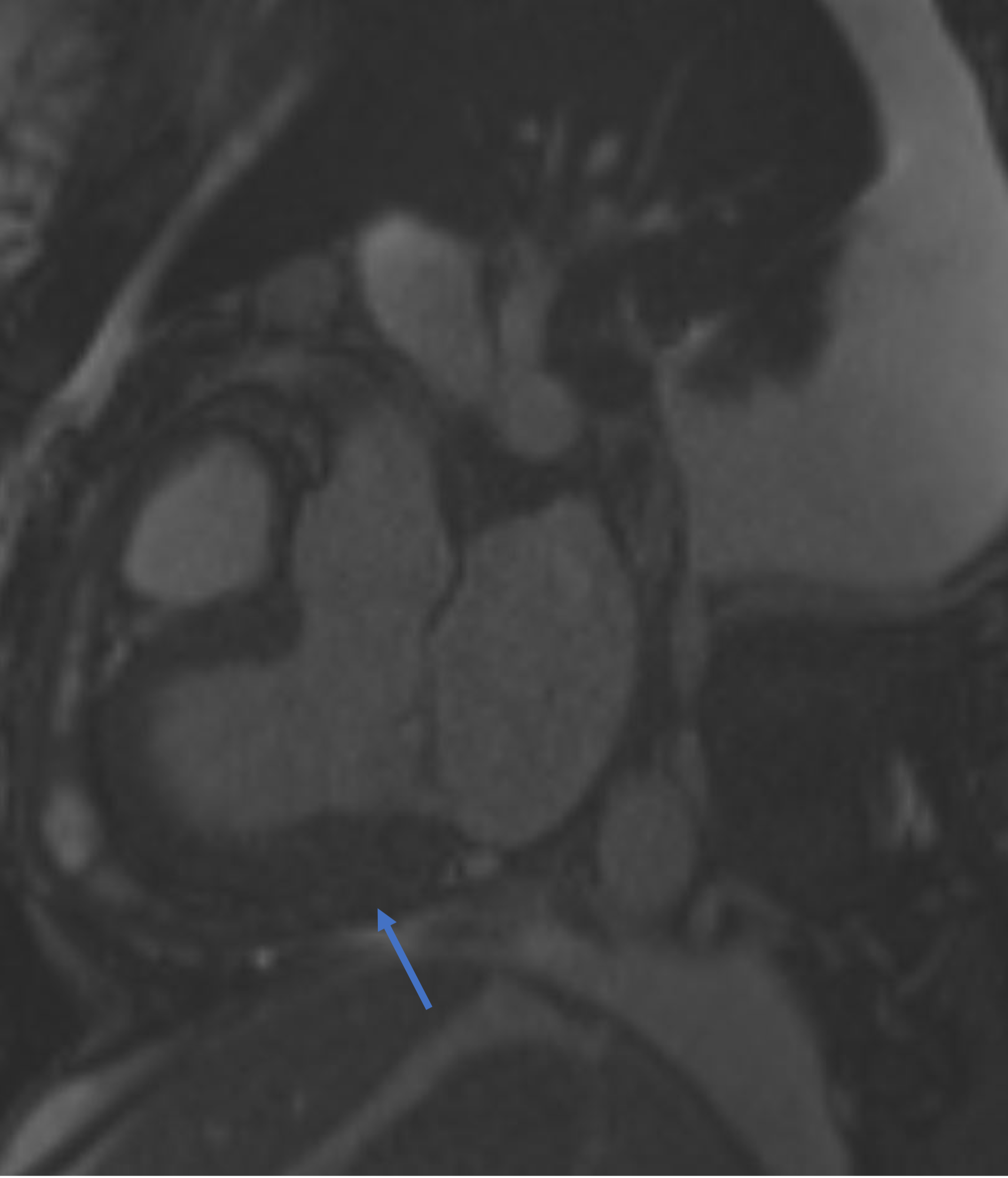

Supplement: ytad540_Supplementary_Data [file ytad540_supplementary_data.zip › Sup Fig 3.png]

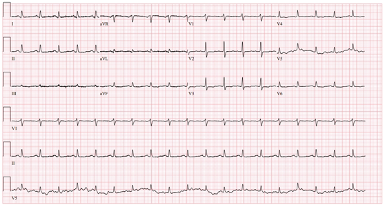

Supplement: ytad540_Supplementary_Data [file ytad540_supplementary_data.zip › Sup Fig 4.png]

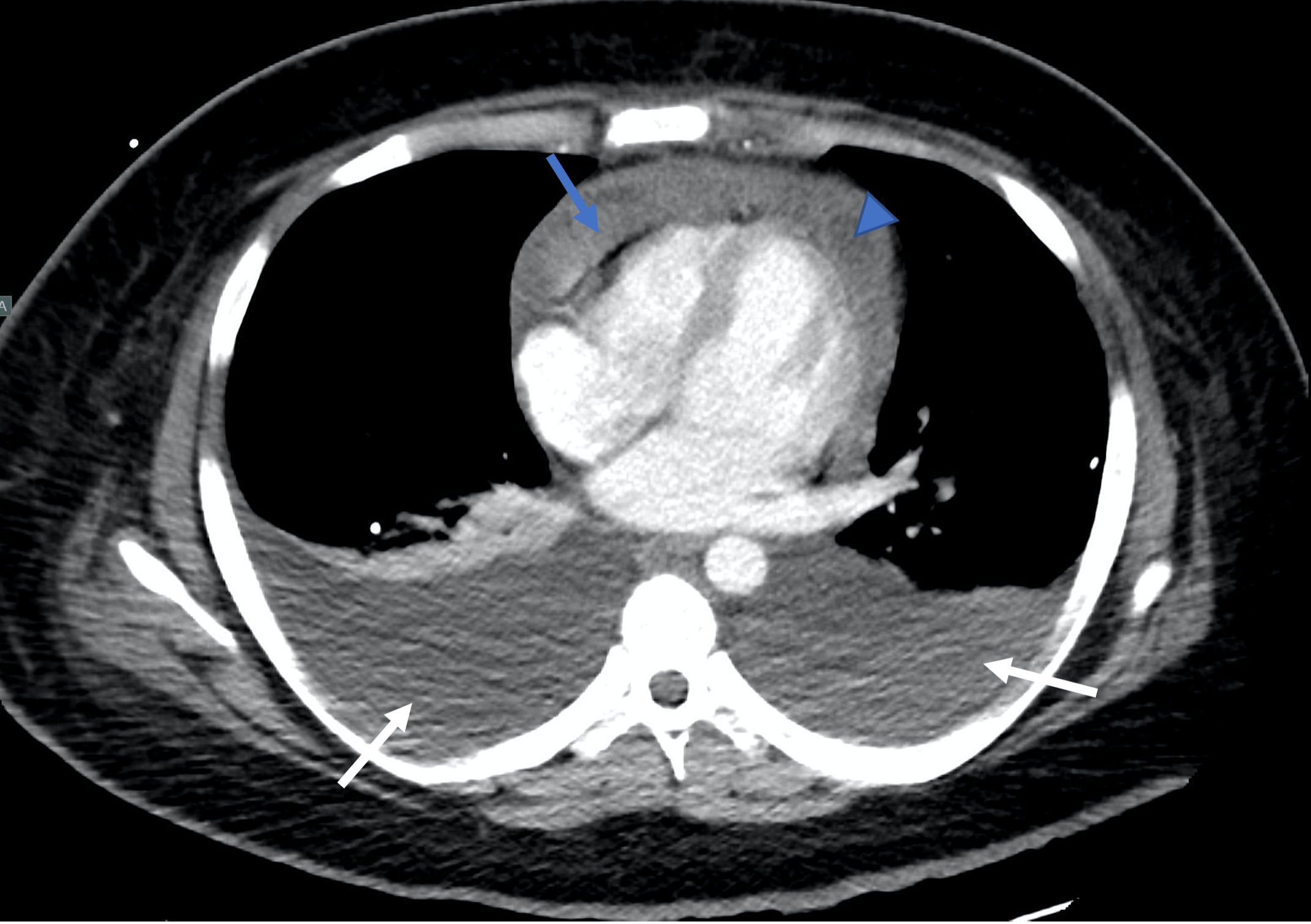

Supplement: ytad540_Supplementary_Data [file ytad540_supplementary_data.zip › Sup Fig 5.png]

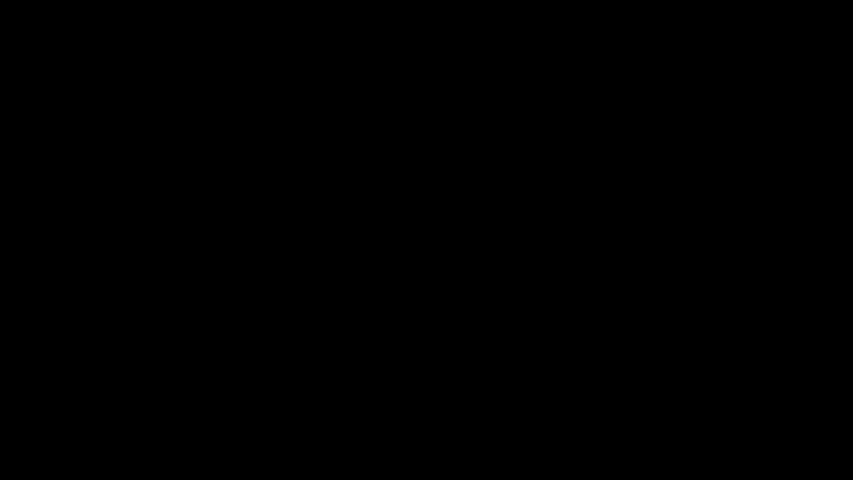

Supplement: ytad540_Supplementary_Data [file ytad540_supplementary_data.zip › CPCIne.gif]

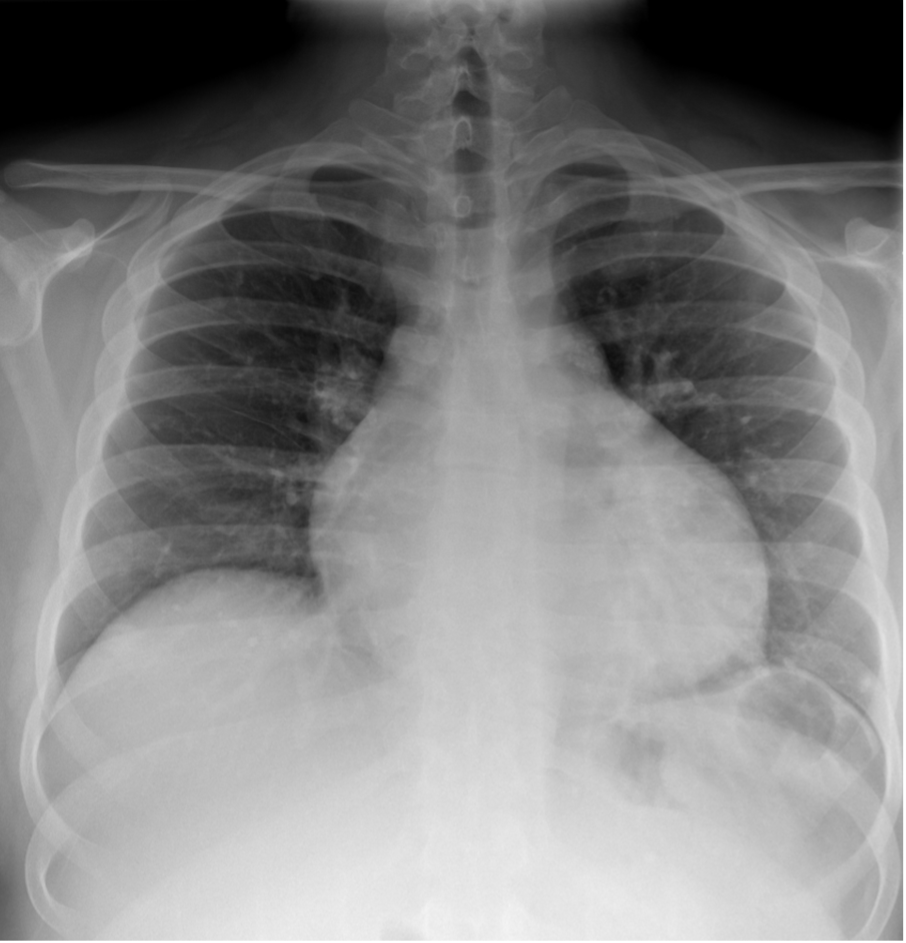

Supplement: ytad540_Supplementary_Data [file ytad540_supplementary_data.zip › Sup Fig 1.png]
